# TERC promotes cellular inflammatory response independent of telomerase

**DOI:** 10.1093/nar/gkz584

**Published:** 2019-07-11

**Authors:** Haiying Liu, Yiding Yang, Yuanlong Ge, Juanhong Liu, Yong Zhao

**Affiliations:** 1 MOE Key Laboratory of Gene Function and Regulation, School of Life Sciences, Sun Yat-sen University, Guangzhou 510006, P.R. China; 2 Department of Endocrinology, Tongji Hospital, Tongji Medical College, Huazhong University of Science and Technology, Wuhan, Hubei 430030, P.R. China

## Abstract

TERC is an RNA component of telomerase. However, TERC is also ubiquitously expressed in most human terminally differentiated cells, which don’t have telomerase activity. The function of TERC in these cells is largely unknown. Here, we report that TERC enhances the expression and secretion of inflammatory cytokines by stimulating NK-κB pathway in a telomerase-independent manner. The ectopic expression of TERC in telomerase-negative cells alters the expression of 431 genes with high enrichment of those involved in cellular immunity. We perform genome-wide screening using a previously identified ‘binding motif’ of TERC and identify 14 genes that are transcriptionally regulated by TERC. Among them, four genes (LIN37, TPRG1L, TYROBP and USP16) are demonstrated to stimulate the activation of NK-κB pathway. Mechanistically, TERC associates with the promoter of these genes through forming RNA–DNA triplexes, thereby enhancing their transcription. *In vivo*, expression levels of TERC and TERC target genes (TYROBP, TPRG1L and USP16) are upregulated in patients with inflammation-related diseases such as type II diabetes and multiple sclerosis. Collectively, these results reveal an unknown function of TERC on stimulating inflammatory response and highlight a new mechanism by which TERC modulates gene transcription. TERC may be a new target for the development of anti-inflammation therapeutics.

## INTRODUCTION

Human telomerase RNA component (TERC) is a 451 nt long, noncoding RNA (lncRNA) that is an essential component of telomerase. TERC serves as a template for reverse transcriptase TERT, which adds GGTTAG repeats to chromosome ends (1). TERC is expressed in all telomerase positive cells, including human stem cells and most cancer cells (2,3). However, TERC is also ubiquitously expressed in terminally differentiated human somatic cells, which do not express TERT and therefore have no telomerase activity (4). The function of TERC in these cells remains largely unknown. In telomerase positive cells, the level of TERC is often higher than that required to form telomerase with TERT (5,6). For telomerase negative cancers, termed alternative lengthening of telomeres (ALT) cancers, TERC is often detected, but TERT is lacking (7). TERC has been considered for a long time as a nonfunctional RNA waiting for TERT to form active telomerase. However, recent discoveries hypothesized that TERC may play a role beyond telomerase. For instance, Gazzaniga *et al.* found that TERC has telomerase independent anti-apoptotic functions in human T cells (8). In addition, it has been reported that TERC is involved in regulating ATR-mediated DNA damage signals and in activation of DNA-PKcs that phosphorylates hnRNP A1 in a telomerase independent manner (9,10).

LncRNAs may regulate gene expression in different ways. For instance, lncRNAs can modulate the epigenetic status of target genes, influencing their transcription (11). In addition, lncRNA may up- or downregulate gene transcription in *cis* by associating with their promoters (12). In this scenario, recent studies found that lncRNA-chromatin interaction is highly sequence dependent with many ‘binding motifs’ exiting in lncRNA (13). It is thus proposed that using its binding motif, lncRNA may hybridize with targeted genes and regulate their transcription. Indeed, it has been demonstrated that many lncRNA–DNA interactions are mediated by formation of RNA–DNA triplexes (14–16). Although the ‘binding motif’ of TERC was identified years ago and it is hypothesized that as a typical lncRNA, TERC may participate in the regulation of gene transcription, whether and how many genes are transcriptionally regulated by TERC still remained elusive.

Here, we reported a new function of TERC as an lncRNA. It stimulates the NF-κB pathway and increases the expression and secretion of inflammation cytokines. By screening the genome for potential TERC binding sites in promoters, we identified 30 genes, of which four (LIN37, TPRG1L, TYROBP and USP16) were validated to be transcriptionally regulated by TERC. Interestingly, these four genes, including three first defined, were related to activation of NF-κB pathway and cellular inflammation. In addition, *in vivo* study showed that expression level of TERC as well as its downstream genes TYROBP, TPRG1L and USP16 were upregulated in patients with chronic inflammation disease.

## MATERIALS AND METHODS

### Reagents

Antibodies to phosphorylated STAT3, STAT3, phosphorylated p65 and p65 were purchased from Beyotime (Shanghai, China). The AKT inhibitor perifosine, NF-κB cofactor IκB inhibitor bay11-7082, and STAT3 inhibitor Stattic were from EFEbio (Shanghai, China). LPS was from Sigma. TNF-α was from PeproTech. ELISA kits for cytokines were purchased from 4A Biotech (Beijing China).

### Cell culture, vectors, and transfections

Cells were cultured in Dulbecco's modified Eagle's medium (Gibco) with 10% fetal bovine serum, 100 U/ml penicillin and 100 μg/ml streptomycin. To generate TERC stably overexpressing cell line, 293T cells were transfected with pBabe-TERC or empty pBabe plasmid and the retroviral packaging plasmids pCMV-VSV.G and pCMV-Gag-Pol (Addgene) using calcium phosphate precipitation. The viral supernatants were collected 72 h after transfection, ultracentrifuged at 40 000 rpm for 2h at 4°C, and then used to infect U2OS cells. Forty-eight hours later, cells were selected with 2 μg/ml puromycin for 3 days. The retained cells were cultured in 1 μg/ml puromycin to produce a polyclonal cell population. The NF-κB luciferase plasmid was a gift from Prof. Jun Cui at Sun Yat-sen University. Plasmid contains a firefly luciferase gene driven by minimal TATA promoter with NF-κB response elements.

SiRNAs were used to knock down genes. SiRNA transfection was carried out with Lipofectamine RNAiMAX (Invitrogen) according to the manufacturer's instructions. Experiments were performed 72 h after siRNA transfection. The sequences of siRNAs are as follows: TERC si1: GUCUAACCCUAACUGAGAAGG; TERC si2: CCGUUCAUUCUAGAGCAAAC; Lin37 si1: GCAGCGAUCCAACACAUAU; Lin37 si2: CCAACACAUAUGUGAUCAA; SLC26A1 si1: GCAACACCCAUGGCAAUUA; SLC26A1 si2: GCCUCUAUACGUCCUUCUU; TPRG1L si1: CCAUUUCCUACGGAGAAUU; TPRG1L si2: GGAAUCCCUGGUCUACCAA; TYROBP si1: GGUGCUGACAGUGCUCAUU; TYROBP si2: UCCUUCACUUGCCUGGACG; USP16 si1: CCAUGAGCCAGUUUCUUAA; USP16 si2: GCAGAUGCUAAUUUCUCUU;

### RNA sequencing and data analysis

The RNA sample preparation, sequencing and data analysis were performed as previously reported (17). The genes with log_2_-fold change ≥2 or ≤–2 as well as FDR<0.01 were considered up- or downregulated genes, respectively. The Gene Ontology and KEGG pathway analyses were performed for upregulated genes in TERC-U2OS cells using the online tool DAVID (18,19).

### Potential TERC binding site search in the promoter database

Promoter sequences of *Homo sapiens* were downloaded from Eukaryotic Promoter Database (https://epd.vital-it.ch/index.php) (20). Promoters containing at least 10 continuous nucleotides of ‘GGCCACCACCCC’ or its reverse complement sequence were defined as potential binding sites of TERC.

### ChIRP-PCR assay

The ChIRP pull down assay was performed exactly as previously reported (13,21) using odd probes of TERC and lacZ. The product was detected by PCR with primers targeting promoters containing potential TERC binding sites. The potential target genes and PCR primer sequences are as follows: Lin37: F 5’-TTGGTCAGGATGCGAGATT-3’, R 5’-TCCTCCGCCTTTGGTTGT-3’; TPRG1L: F 5’-GCAAGGCGGAGCCAATCG-3’, R 5’-ACCCCTTACCGACCCCGAC-3’; TYROBP: F 5’-CAAGTGAAGGAGGAAGTCTGA-3’, R 5’-CCTGATTCTTTCTTGGGTTTT -3’; USP16: F 5’-TCAGAGCCGATGGTCCCG -3’, R 5’- CTCCGTCTTCCTCCTGGTGA -3’.

### Electrophoretic mobility shift assay

Oligonucleotides were synthesized (Generay, China) and annealed into dsDNA in binding buffer (10 mM Tris–HCl pH7.4, 125 mM NaCl, 6 mM MgCl_2_). Oligonucleotides were heated at 95°C for 3 min and cooled down to 50°C with every 5°C interval (90, 85, 80°C...) helding for 3 min. *In vitro* TERC transcription was performed using High Yield Transcription Kit (Ambion), 450 nt Scramble sequence was used as a control. 1pmol of dsDNA was incubated with different amounts of TERC in binding buffer for 2 h at 42°C. The samples were analyzed on 2% agarose gel at 4°C.

### Melting profiles

The ds-promoter segments of LIN37, TPRG1L, TYROBP and USP16 (in Table 1) were formed and mixed with or without TERC or TERC1–49nt at the ratio of 1:1 (mole). The melting profile was obtained in q-PCR working buffer containing SYSB green I in LightCycler 480 (Roche). Samples were incubated at 60°C for 1 min, and then increased to 95°C at the rate of 2.5°C/s. Fluorescence intensity was detected at 0.2°C interval.

### ELISA and Western blotting

For detection of cytokine secretion, U2OS cells were treated with 10 ng/ml TNF-α, whereas B2-17 cells were treated with 1 μg/ml LPS for 6 h. The culture medium was collected and cytokines were detected using corresponding ELISA kits. For Western blotting, U2OS cells were treated with or without TNF-α for indicated times. To inhibit NF-κB or STAT3 pathway, U2OS cells were treated with indicated inhibitors for 6 h. The culture medium was collected for ELISA analysis, and the cells were collected for Western blotting.

### TERC expression in clinical samples

Processed gene expression profiles of clinical samples (GSE54350, GSE65561 and GES66988) were downloaded from GEO database (Gene Expression Omnibus). Expression levels of TERC and its target genes were analyzed.

### Mapping of TERC-ChIRP fragments to genome

TERC CHIRP-seq dataset was downloaded from published article (13). The original dataset included 2198 fragments located in hg18. These fragments were re-localized in hg19. The nearest transcription start sites (TSSs) were identified. The distance between fragment and TSS was calculated and defined as “distance to TSS".

### Quantitative RT-PCR

Total RNA was isolated using TRIzol (Takara). Approximately 1 μg RNA per sample was used to generate cDNA by reverse transcription. Real-time PCR was carried out with q-PCR buffer containing SYBR Green I in LightCycler 480 (Roche). All PCR primer sequences were from PrimerBank (22).

### Luciferase reporter assays

HEK293T cells were plated in 96-well plates (  1×10^4^ per well) and transfected with plasmids encoding NF-κB luciferase reporter (firefly luciferase plasmid), pRL-TK-luc (renilla luciferase plasmid) and pBabe-TERC (Full-length TERC). For knockdown experiment, siTERC was transfected into HEK293T cells. Cells were harvested at 48 h after transfection and luciferase activity was measured with Dual-Luciferase Assay kit (Promega) according to the protocol provided by manufacturer. The firefly luciferase intensity of NF-κB reporter was first normalized to renilla luciferase and then divided by corresponding control to obtain a relative activity of NF-κB luciferase (Folds).

### Immunofluorescence (IF)

Cells were grown on coverslip, washed with PBS and fixed in 4% paraformaldehyde for 15 min at room temperature, and then permeabilized in 0.5% Triton X-100 at room temperature for 30 min. The cells were washed thrice with 1× PBST and blocked with 5% goat serum for 1 h at room temperature. The cells were incubated sequentially with anti-p65 antibody overnight at 4°C and secondary antibody conjugated with DyLight 488 for 1 h at room temperature. The coverslip was washed with PBST, mounted with DAPI, and visualized using a Zeiss microscope.

## RESULTS

### TERC promotes cellular inflammatory response

**Figure 1.**
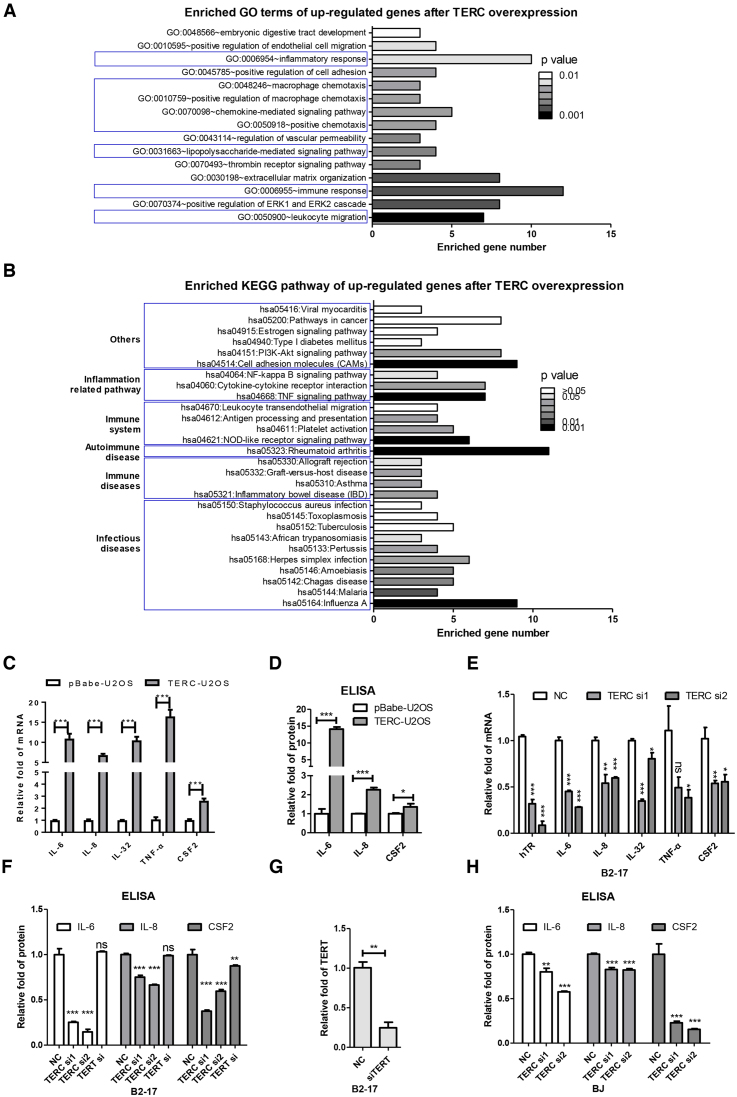
TERC promotes inflammatory response. (**A**) Enriched biological processes after TERC overexpression. Gene ontology was analyzed for upregulated genes in TERC-U2OS compared to pBabe-U2OS. GO terms in blue boxes were immune related biological processes. (B) Enriched signaling pathways after TERC overexpression. KEGG pathway enrichment was analyzed with upregulated genes in TERC-U2OS compared to pBabe-U2OS. Different categories of pathways were boxed and labeled. (**C**) The mRNA levels of cytokines after TERC overexpression in U2OS cells. The stable cell lines pBabe-U2OS and TERC-U2OS were stimulated with TNF-α for 1 h, and cells were collected for qPCR. (**D**) The secreted cytokines after TERC overexpression in U2OS cells. The stable cell lines pBabe-U2OS and TERC-U2OS were stimulated by TNF-α for 6 h, and culture medium was collected for ELISA. (**E**) The mRNA levels of cytokines after TERC knockdown in B2–17 cells. The cells were transfected with indicated siRNAs for 72 h and treated with LPS during the last 1 h of transfection. Cells were then collected for qPCR. (**F**) The secreted cytokines in B2–17 cells after TERC or TERT knockdown. Cells were transfected with indicated siRNAs for 72 h and treated with LPS during the last 6 h of transfection. Culture medium was then collected for ELISA. (**G**) The knockdown efficiency of TERT. The B2–17 cells were transfected with TERT siRNAs for 72 h, and cells were collected for qPCR. (**H**) The secreted cytokines in BJ after TERC knockdown. Cells were transfected with indicated siRNAs for 72 h and treated with TNF-α during the last 6 h of transfection. Culture medium was collected for ELISA. All values are means ± SEM of more than three independent experiments (*P< 0.05, **P< 0.01,***P< 0.001).

To explore the biologic functions of TERC beyond serving as a template for telomerase extension, we ectopically expressed TERC in telomerase negative U2OS cells in which endogenous TERC was in the minutest amount and TERT was undetectable. Differentially expressed genes were then examined by RNA-seq. The result showed that 431 genes were up- or downregulated with log_2_-fold change ≥2 or ≤–2, respectively (Supplementary Table S1). Upregulated genes were then subjected to Gene Oncology (GO) analysis. Strikingly, 8 of 15 enriched GO terms were related to immunology, including immune response, chemokine-mediated signaling pathway, macrophage chemotaxis, and inflammatory response (Figure 1A). Alternatively, when upregulated genes underwent KEGG pathway analysis, 22 out of 28 KEGG terms were related to immunology such as immune system, immune related diseases or inflammation related pathway (Figure 1B). These results strongly suggested that TERC is involved in cellular immune in a direct or indirect manner.

To further explore this hypothesis, we stimulated TERC-U2OS and control pBabe-U2OS cells with TNF-α and detected the expression and secretion of inflammatory factors. The results showed that mRNA levels of five cytokines (IL-6, IL-8, IL-32, TNF-α and CSF2) were upregulated in TERC-U2OS compared to pBabe-U2OS (Figure 1C). Accordingly, secreted cytokines (IL-6, IL-8 and CSF2) in culture medium were also increased (Figure 1D). Conversely, when TERC was depleted by siRNAs in human astrocytoma B2–17cells (siRNA with a scramble sequence was used as a control), both the expression and secretion level of cytokines significantly decreased (Figure 1E and F). Because B2–17 are telomerase positive cells, to exclude the engagement of telomerase in the inflammatory response, TERT was knocked down (Figure 1G) and cytokine secretion was determined. Our results showed that in contrast to knockdown of TERC, depletion of TERT only slightly decreased the secretion of CSF2 and had no effect on IL-6 and IL-8 (Figure 1F). To further confirm the role of TERC in the inflammatory response, TERC was knocked down in human normal fibroblast BJ cells, which do not express TERT and therefore do not have telomerase activity. We observed that depletion of TERC significantly decreased secretion of IL-6, IL-8 and CSF2 in response to immune stimulation (Figure 1H).

### Activation of the NF-κB signaling pathway by TERC

**Figure 2.**
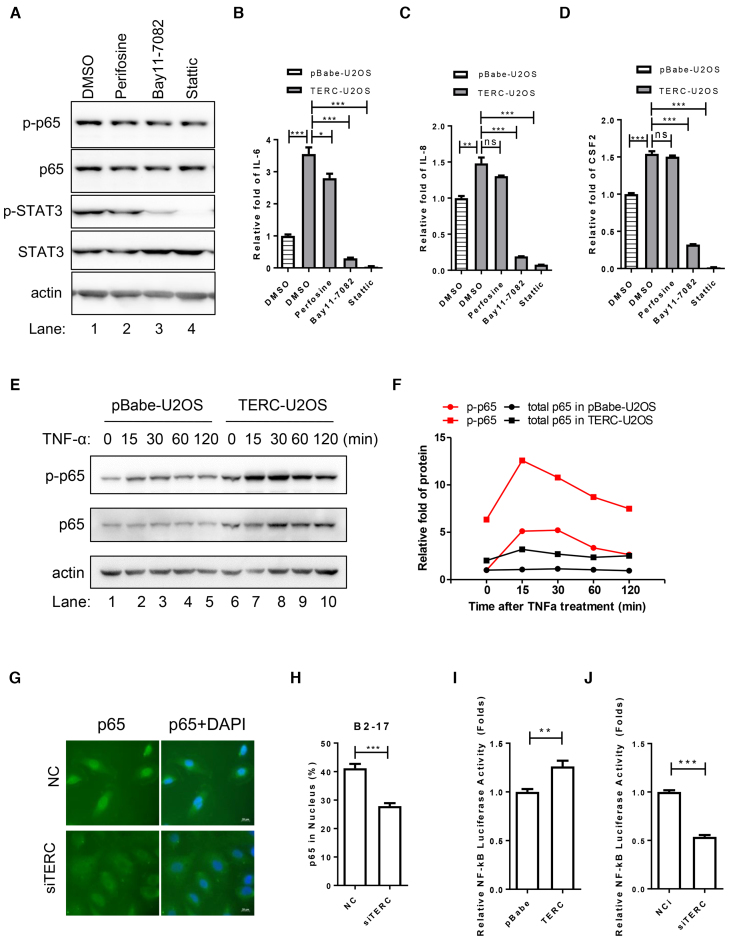
TERC activates the NF-κB pathway. (**A**) Inhibition of NF-κB or STAT3 activation by inhibitors in TERC-U2OS. TERC-U2OS cells were treated with inhibitors of AKT, IκB and STAT3 (Perifosine, Bay11–7082 and Stattic, respectively) for 6h, and cells were collected for Western blotting of indicated proteins. (**B-D**) The TERC promotion of cytokine secretion was counteracted by inhibitors of p65 cofactors IκB and STAT3. Stable cell lines pBabe-U2OS and TERC-U2OS were treated with TNF-α plus indicated inhibitor for 6 h, and culture medium was collected for IL-6, IL-8 and CSF2 detection by ELISA. (**E**) Total and phosphorylated p65 were upregulated by TERC. PBabe-U2OS and TERC-U2OS cells were treated with TNF-α for indicated times. Total and phosphorylated levels of p65 were determined by Western blotting. (**F**) Quantification of (E). Protein levels in (E) were quantified and normalized by actin. (**G**) Inhibited nuclear translocation of p65 in TERC depleted cells. B2–17 cells were transfected with TERC siRNA or NC for 72 h and then treated with TNF-α (10 ng/ml) for 20 min. Immunofluorescence (IF) using p65 antibody was performed to determine the amount of p65 in nucleus and cytoplasm. (**H**) Quantification of (G). Nuclear p65 (Fluorescence intensity) was quantified as a percentage of overall p65 in cell. ∼100 cells were counted for each experiment. (**I**, **J**) NF-κB luciferase reporter assay for analysis of activation of NF-κB in TERC overexpressed (I) and depleted cells (J). HEK293T cells were transfected with TERC/pBabe (I) or siTERC/NC (J) together with NF-κB-luc and pRL-TK. Luciferase activity was determined 48 h after transfection. All values are means ± SEM of more than three independent experiments (*P < 0.05, **P< 0.01, ***P< 0.001).

The NF-κB signaling pathway plays a fundamental role in inflammatory response, governing the release of inflammatory factors (23). We noticed that NF-κB signaling pathway was enriched by KEGG pathway analysis in TERC overexpressing cells (Figure 1B). To explore whether TERC-induced stimulation of inflammatory response is mediated by NF-κB pathway, inhibitors of p65 cofactors IκB and STAT3 (Bay11-7082 and Stattic, respectively) were used to treat TERC-U2OS. Indeed, both Bay11-7082 and Stattic, but not the AKT inhibitor perifosine, suppressed the increase of IL-6, IL-8 and CSF2 induced by TERC overexpession (Figure 2A–D).

Furthermore, we observed that NF-κB subunit p65 and phosphorylated p65 (p-p65) were higher in TERC-U2OS than in control pBabe-U2OS, suggesting the activation of the NF-κB pathway (Figure 2E, 0min). When cells were further stimulated with TNF-α, the level of p65 and p-p65 in TERC-U2OS were continuously higher than pBabe-U2OS throughout the activation procedure, suggesting that TERC stimulates activation of NF-κB pathway (Figure 2E, F). Upon activation of NF-κB pathway, p65 migrates from cytoplasm to nucleus where it functions to initiate the transcription of target genes (24). The amount of p65 in nucleus is indicative of activation extent of NF-κB pathway. We observed that in response to TNF-α treatment, TERC-depleted cells display much less p65 in nucleus than control cells, demonstrating that TERC positively regulates the activation of NF-κB pathway (Figure 2G, H). Moreover, we also performed luciferase reporter assay, in which NF-κB response element is placed in TATA promoter that drives the transcription of luciferase. The result showed that compared to control cells, overexpression of TERC increases luciferase activity, whereas knockdown of TERC decreases its activity (Figure 2I and J). Altogether, these data confirmed that TERC promotes the activation of NF-κB pathway.

### TERC regulates gene transcription by targeting its promoter

**Figure 3.**
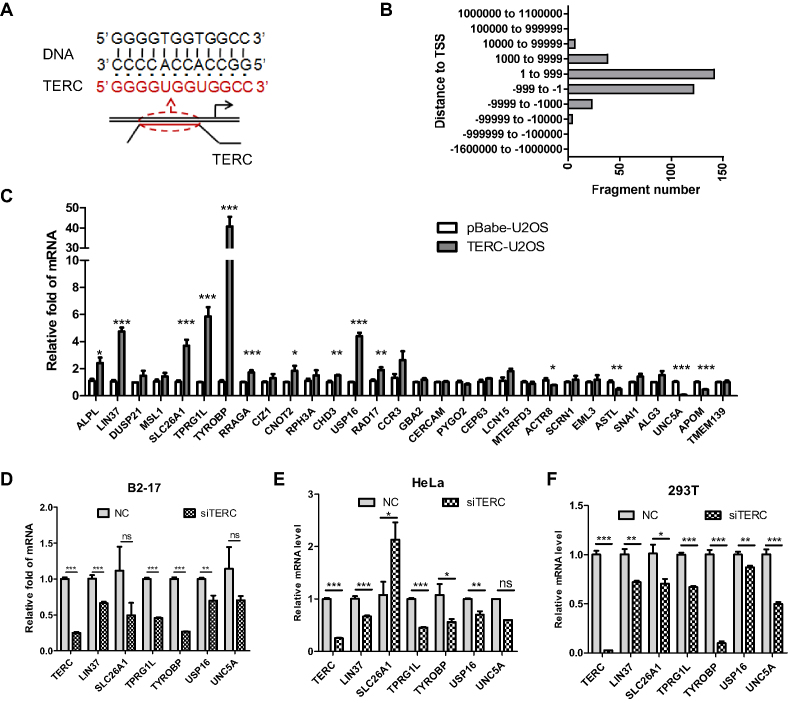
TERC targets gene's promoters using its binding motif. (**A**) Schematic diagram of TERC targeting to gene's promoters that contain the sequence of TERC binding motif. (**B**). Distribution of TERC targeted fragments on genome. The fragment was classified according to its distance to the nearest TSS. (**C**) The mRNA levels of 30 genes with TERC binding motif in their promoters in TERC-U2OS *vs* pBabe-U2OS cells. (**D–F**). Determination of mRNA levels of top 6 genes in (C) that have the highest fold change (up- and downregulation) in response to TERC expression. Gene expression was tested in indicated cells with or without TERC knockdown. Each panel represents different cells as indicated. All values are means ± SEM of more than three independent experiments (*P< 0.05, **P< 0.01, ***P< 0.001).

We next explored how TERC stimulates the NF-κB pathway. As a typical lncRNA, we speculated that TERC may regulate gene transcription by targeting specific sites in the genome. Indeed, using chromatin isolation by RNA purification (ChIRP), it was previously reported that TERC targets the genome with a high preference for the sequence 5′-GGCCACCACCCC-3′ (termed the binding motif) (13), which is exactly complementary to the sequence 5′-GGGGUGGUGGCC-3′ at site 25–36 of TERC. This strongly suggested that TERC binds to genomic DNA using its binding motif, which may form a triplex structure with target DNA (Figure 3A).

To survey the locations of TERC target sites, we downloaded 2198 TERC-ChIRP fragments from published study (13) and mapped them to the human genome. The distance to the nearest transcription start site (TSS) of each fragment was calculated. Most fragments (TERC targeted sites) were located within ±1000 bp of TSSs (Figure 3B), suggesting that TERC tends to target gene's promoters. Then, we screened potential binding sequences using a TERC binding motif in Eukaryotic Promoter Database (EPD) (20), which contains all identified promoters and sequences adjacent to TSSs. As a result, as many as 30 sequences on gene promoters were identified that were listed in Table 1. We then verified whether the transcription of these genes was altered by TERC using pBabe-U2OS and TERC-U2OS cells. 14 of 30 genes exhibited up- or downregulated transcription in response to TERC expression (Figure 3C). The top six up- or downregulated genes were LIN37, SLC26A1, TPRG1L, TYROBP, USP16 and UNC5A. Among them, four genes (LIN37, TPRG1L, TYROBP and USP16) showed consistent downregulation when TERC was knockdown in B2–17, HeLa and 293T cells (Figure 3D–F). We thus focused on these four genes.

**Table 1.** Promoters containing potential TERC binding site

Gene Name	Gene Promoter Seq. from EPD
ALPL	actcgggccccgcggccgcctttataaggcggcgggggtggtggcccggGCCGCGTTGCG
LIN37	taataggaacaagctactgccgaaggggcccgcccacagaagggtggtgGCCACGGTCCA
DUSP21	agatgggagtggtgagaggagacagaaagagggtggtggccgatagctgGTCCTCTTTCT
MSL1	gcggccagcgagggcagatggaagagTATGAGGAAGAGCCCTCTCGGGGGTGGTGGCGGC
SLC26A1	cagagtccagggcacagaccactgcctgcaggttggcgccaccacccccACTCTCCCCGC
TPRG1L	ccgcggggcggggccgggggcgcggccgggtggtggcggtggctgcggcgacggcggtcG
TYROBP	ccctgtctcctcctcccttctgccaccacccgcctcagacttcctccttCACTTGCCTGG
RRAGA	gagatcgccgccggaagtgggtggtggcggggacgcagcggctccctccCGGAAAGCGAG
CIZ1	ggcaaaatggcgaaatccctctctacaaaaaatacaaacattagccagggtggtggcggg
CNOT2	agggagggagggggtgtgtatgggggtggtggtggACCGGACGTAAAGCGTCGCTGTACT
RPH3A	aggagggagagggggtggtggaggagggagagagggtgggagaaggagtgatgaagatgg
CHD3	ggggaggcgggcgggcggtgggtgggggggtggtgggggggccAGAGCCACAGGATGGCT
USP16	GGGAGGTGGGGGTGGGGTGGTGGTGGCCTAGCCACTTCCCATAATGCCGCGTTCCGGAAG
RAD17	CGAATATTTGAGCTTAGTATTCCCTGTTCACTGTGTGGGGTGGTGGTGGGTCGGCTAGGA
CCR3	aggtggtggcctgcccctccccgcaggcactctgtcccagggagaaatcagaactcttta
GBA2	CACGGCCACTTCTGCATCCAGGTGGGGATGCTGGCACTGAAGGTGGTGGCCCTTCTGGGA
CERCAM	GGAGCCGGGGAAGCCCGGGAGGTGGTGGCCGAGTGGGCGCCGCCCCTCTGGGTCTGCGGC
PYGO2	ttgctccccctccccgcagcgctcagtggtggtggccgcgacgagttccGGTTCCGGTTG
CEP63	GCCTCGCAGGCCACCACCATCCGCACCGTACGACAGGCCGTCCCTCAGCTGCGGCTTCCT
LCN15	caggtggtggcctgggctataaagctggccccctggggcttggggactcAGCACCAGGGG
MTERFD3	ggaagcaaatgcagctggtgcaggagagggaaatgggaattagggtggtGGCAGAGCCCA
ACTR8	cccctggtggggggAGTGCGGAAGCGGTCGTTCTTTTCCGGGTGGTGGCGCGCCGGGACG
SCRN1	tcccactcctctccacctccactgccaccaccctgcaccaagccaccaccatctccagcc
EML3	CTCGGGGTGGTGGTACGGCGCCCTTCGCGCGCGCCCCGGGGTGCTTCCCCTTCCCCTCTC
ASTL	GTAACCTAATTGCAGAACCGGCACCACCACCCCCTCTTAAATAGCAGCTGctccacctcc
SNAI1	CCACCACCCCCCCGGAGTACTTAAGGGAGTTGGCGGCGCTGCTGCATTCATTGCGCCGCG
ALG3	aagcggaacctaagtgtcgaaggttcgggtttccgggggtggtgggcccACACAAGCGGC
UNC5A	GCCCACCACCCCAAGCCCCTCCCTGGGGGAGCCTCAGGCATCGCCCAGAGGGATTCCCGG
APOM	acacacccaccaccccgcggctccgcccccgacttccccacggaccgtcACTTCCGGTCT
TMEM139	acctacccgctccggcccttcccaccaccccccaccccatctactttctACAGTCTGTGG

Underline indicates potential binding sites

Lower case indicates upstream of TSS

### TERC binds to gene's promoters by forming RNA–DNA triplexes

**Figure 4.**
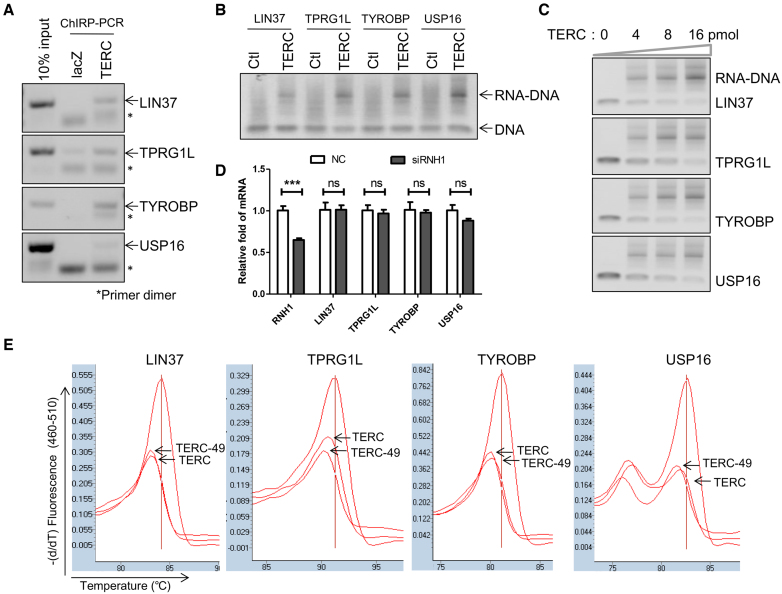
RNA–DNA triplex formation between TERC and gene's promoters. (**A**) TERC binds to the promoter of indicated gene *in vivo*. TERC ChIRP was performed using TERC probe. LacZ probe was used as control. Promoters of the indicated genes were detected by PCR. (**B**) TERC binds to the promoters of indicated genes *in vitro*. EMSA was performed to detect the triplex formation between synthesized gene promoters containing TERC binding motif and TERC. RNA with random sequence was used as control. Products were analyzed on 2% agarose gels. (**C**) Triplex formation in a dose dependent manner. Two picomoles of indicated gene promoters were incubated with increasing amounts of TERC. Products were analyzed on 2% agarose gels. (**D**) Expression levels of indicated genes after RNH1 knockdown. U2OS cells were transfected with siRNH1 for 72 h, and corresponding mRNAs were detected by qPCR. (**E**) Melting temperature decreased after triplex formation at neutral pH. Melting temperatures of gene's promoters were detected in the presence or absence of TERC or TERC1–49. All values are means ± SEM of more than three independent experiments (*P< 0.05, **P< 0.01, ***P< 0.001).

To investigate whether TERC binds to promoters of LIN37, TPRG1L, TYROBP and USP16 *in vivo*, ChIRP-PCR was performed, in which biotin-labeled oligonucleotides were used to pull down TERC and associated chromatin (13,21). PCR was then performed using primers that cover the TERC binding site in the gene's promoter (Table 1). The results showed that promoters of four genes were enriched by ChIRP, demonstrating association of these promoters with TERC (Figure 4A).

To further validate the interaction between TERC and promoters, electrophoretic mobility shift assay (EMSA) was carried out *in vitro* by incubating TERC and synthesized 59 bp double-stranded (ds) DNA that is from indicated gene promoter and consists of binding motif sequence of TERC. Indeed, shifted bands were observed for all four tested promoters, but not for scramble sequence (Figure 4B). In addition, with increased TERC, shift bands (RNA–DNA) gradually increased and free promoter DNA decreased accordingly (Figure 4C).

TERC may form R-loop or triplex with targeted ds-promoters. To test the possibility that TERC may form R-loops by hybridizing with targeted DNA, we knocked down the RNH1 gene in TERC-U2OS cells. RNase H is an enzyme that digests RNA in Watson–Crick RNA–DNA hybrids (25). It is speculated that gene expression levels should be altered by either overexpression or knockdown of RNase H if they are regulated by R-loop (26). In contrast to this, we observed no change in the expression levels of four genes when RNase H was knocked down (Figure 4D). In addition, we observed that melting temperatures of ds-promoters were reduced by approximately 1°C after incubating with TERC or 49 nt TERC fragments that contain the binding motif (Figure 4E). This is consistent with the previous report that melting temperature decreases during conformation change from double-strand DNA into RNA–DNA triplexes (27). Altogether, these results suggested that TERC may target gene's promoters by forming RNA–DNA triplexes.

### TERC-targeted genes activate the NF-κB pathway

**Figure 5.**
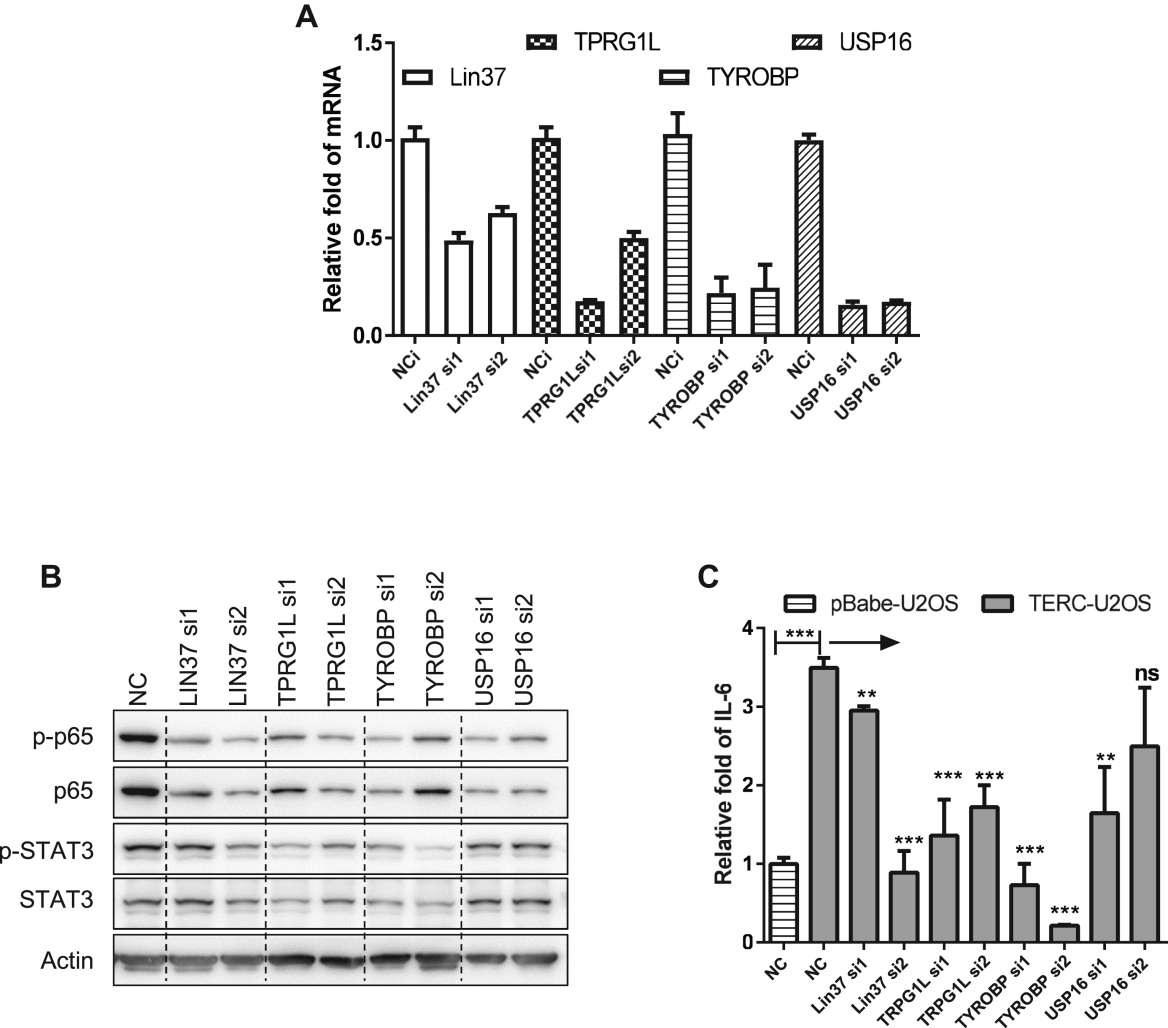
TERC activates NF-κB pathways through targeting immune-related genes. (**A**) The knockdown efficiency of indicated genes. The TERC-U2OS cells were transfected with indicated siRNAs for 72 h, and corresponding mRNAs were detected by qPCR. (**B**) P65 and STAT3 were downregulated by knockdown of TERC targeted genes. The TERC-U2OS cells were transfected with indicated siRNAs for 72 h. Total and phosphorylated p65 and STAT3 expressions were determined by western blotting. (**C**) The TERC promotion of IL-6 secretion was counteracted by knockdown of TERC targeted genes. PBabe-U2OS and TERC-U2OS cells were transfected with indicated siRNAs for 72 h and treated with TNF-α during the last 6 h of transfection. Culture medium was collected for IL-6 detection by ELISA. All values are means ± SEM of more than three independent experiments (*P< 0.05, **P< 0.01, ***P< 0.001).

Because NF-κB component such as p65 or STAT3 is not direct target of TERC, we hypothesized that TERC stimulates the NF-κB pathway in an indirect manner, potentially through promoting the expression of targeted genes. We thus tested identified four genes for their ability to activate the NF-κB pathway. Four genes (LIN37, TPRG1L, TYROBP and USP16) were individually knocked down in TERC-U2OS cells. We found that while knockdown of LIN37, TPRG1L and USP16 decreased the level of p65 and p-p65, the knockdown of LIN37, TPRG1L and TYROBP decreased the level of STAT3 and phosphorylated STAT3 (p-STAT3) (Figure 5A, B). Consistently, ELISA assay showed that knockdown of LIN37, TPRG1L, TYROBP and USP16 counteracted enhanced secretion of IL-6 by TERC (Figure 5C). Altogether, these results demonstrated that TERC modulates the inflammatory response through regulating a group of genes such as LIN37, TPRG1L, TYROBP and USP16. While TYROBP has been previously reported to be involved in the activation of the inflammatory response (28–31), the other three genes are newly identified.

### The expression of TERC and its targeted genes are upregulated in inflammation related disease

**Figure 6.**
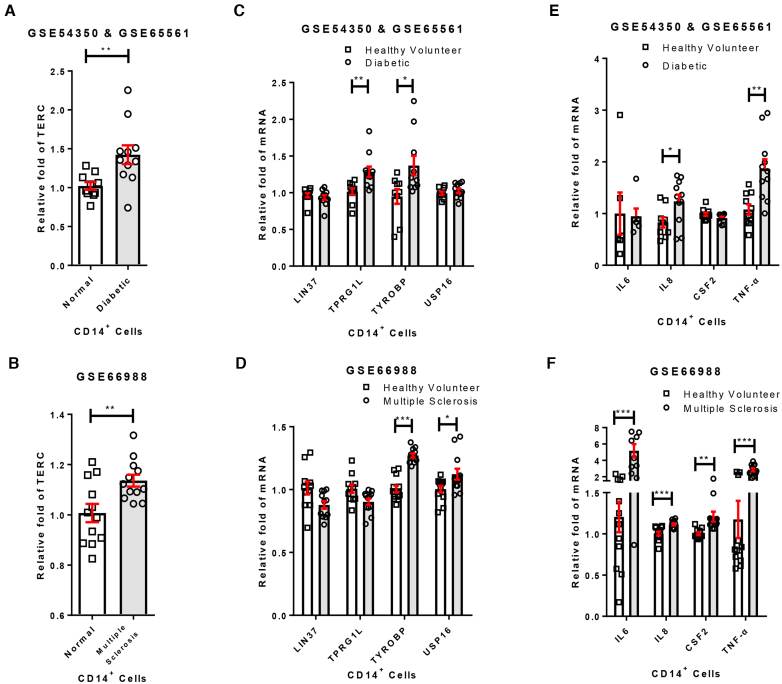
The expression levels of TERC and TERC targeted genes in inflammation related diseases. (**A**) TERC was upregulated in CD14^+^ cells of diabetic patients. Data were downloaded from the GEO database (GSE54350 & GSE65561), and TERC expression levels were analyzed (*n* ≥ 10, *P< 0.05, **P< 0.01). (**B**) TERC was upregulated in CD14^+^ cells of multiple sclerosis patients. Data were downloaded from the GEO database (GSE66988), and TERC expression levels were analyzed (*n* ≥ 10, *P< 0.05, **P< 0.01). (**C**) Expression levels of TERC targeted genes in CD14^+^ cells of diabetic patients. Data analysis is the same as (A). (**D**) Expression levels of TERC targeted genes in CD14^+^ cells of multiple sclerosis patients. Data analysis is the same as (B). (**E**) Expression levels of cytokines in CD14^+^ cells of diabetic patients. Data analysis is the same as (A), except that ‘*n*’ of IL6 is 6 because IL6 is missing in GSE65561. (**F**) Expression levels of cytokines in CD14^+^ cells of multiple sclerosis patients. Data analysis is the same as (B).

Because TERC regulates the expression of inflammation related genes *in vitro*, we then investigated its potential immune-regulation function in patients. First, we analyzed TERC levels in type II diabetes and multiple sclerosis, both displaying increased chronic inflammation (32,33). The expression levels of TERC in CD14+ cells from type II diabetes and multiple sclerosis patients were significantly higher than normal people (Figure 6A and B). We also examined the expression level of LIN37, TPRG1L, TYROBP and USP16 in CD14+ cells from patients and normal people. For diabetic patients, TPRG1L and TYROBP were upregulated, whereas TYROBP and USP16 were upregulated in multiple sclerosis patients (Figure 6C and D). Consistently, the expression of inflammatory cytokines such as IL-8 and TNF-α in diabetic patients and IL-6, IL-8, CSF2 and TNF-α in multiple sclerosis patients increased (Figure 6E and F). Therefore, the elevated inflammatory response is positively correlated with increased expression of TERC and TERC target genes in patients.

## DISCUSSION

A high incidence of TERC mutation and an increased copy number of TERC genes has been reported to associate with the pathogenesis of the inherited disorder dyskeratosis congenital (DC), aplastic anemia (AA) (34,35) and other genetic diseases (36–38). Interestingly, both DC and AA patients also display immune abnormalities (39,40). Moreover, many mutations in TERC do not affect its function as an RNA template and telomerase activity (41–43), implying that TERC may have noncanonical functions beyond telomerase. In this study, we revealed that TERC stimulates cellular inflammatory response in a telomerase independent manner. The evidence supporting this includes: (i) RNA-seq data indicated that expression of immune-related genes are regulated by TERC; (ii) overexpression of TERC in telomerase negative U2OS cells resulted in increased expression and secretion of inflammatory factors; (iii) knockdown of TERC, but not TERT, in telomerase positive cells decreased secretion of inflammatory factors; (iv) overexpression of TERC led to activation of the NF-κB signaling pathway in the absence of allothogenic stimulation; (v) TERC upregulated the expression of LIN37, TPRG1L, TYROBP and USP16 that is linked to the activation of NF-κB signaling pathway, leading to increased inflammatory response and (vi) high TERC levels corresponded to high inflammation states in patients with type II diabetes or multiple sclerosis.

LncRNAs may regulate gene expression in different manners (11). For example, eRNA, which is transcribed from enhancers, promotes chromatin accessibility by remodeling the chromatin (44), whereas lncRNA Khps1, HOTAIR and MEG3 regulate gene transcription by recruiting chromatin modifiers to target sites (13–16). A previous study identified the sequence ‘GGCCACCACCCC’ as a binding motif in TERC that may associate with genomic DNA (13). Here, we found that TERC associated sequences were largely located near the TSSs of genes (Figure 3B). Following this route, we identified 30 potential promoters TERC may bind to. Among them, 4 genes were experimentally verified to be transcriptionally regulated by TERC (LIN37, TPRG1L, TYROBP and USP16). Similar to many other lncRNAs (13–16), we demonstrated that TERC formed triplexes with promoter sequences of these genes and thereby promoted their transcription. It should be noted that identified four genes are representative of 14 genes, which are transcriptionally regulated by TERC (Figure 3). Considering that direct association is one of manners by which lncRNA regulates gene transcription, the total number of genes regulated by TERC might be much greater than 14. Further investigation is thus needed to identify whole genes regulated by TERC.

Except for TYROBP, which was previously reported to engage in inflammatory response, the other three genes are newly identified that could activate the NF-κB signaling pathway. Because p65 and STAT3 are not directly regulated by TERC, we hypothesized that TERC stimulates inflammatory response in an indirect manner, i.e., through transcriptional activation of inflammatory related genes that then activate NF-κB signaling pathway. In the absence of TERC, cells were less sensitive to immune stimulation by TNF-α or LPS (Figure 1F, H). Therefore, we defined TERC as a positive modulator of NF-κB mediated inflammatory response.

It was previously discovered that TERC is implicated in angiogenesis, metastasis and proliferation of cancer cells by regulating the global gene expression (45,46). Similarly, we revealed that TERC promotes cellular inflammatory response by upregulating the expression of immune-related genes such as LIN37, TPRG1L, TYROBP and USP16 in human normal and cancer cells. Moreover, we observed that in patients with chronic inflammation such as Type II diabetes and multiple sclerosis, the expression of TERC is upregulated. Accordingly, the expression of TERC-targeted genes (TPRG1L, TYROBP and USP16) as well as secreted inflammatory factors (IL-6, IL-8, CSF2 and TNF-α) are upregulated, which may contribute to increased inflammatory level in patients. In this context, TERC may be a potential target for anti-inflammatory therapeutics for patients with chronic inflammation.

## CONCLUSIONS

Here, we found that TERC regulates immune-related gene transcription by forming triplex with their promoters. In this way, four genes were identified that promote cellular inflammatory response by enhancing the activation of NF-κB signaling pathway. The axis of TERC/targeted-genes/NF-κB/inflammatory response exists in patients with chronic inflammatory disease.

## DATA AVAILABILITY

The raw RNAseq data of pBabe-U2OS and TERC-U2OS (hTR-U2OS in the website) have been submitted to GEO database. The GEO accession number is GSE125024. The link of the dataset is: https://www.ncbi.nlm.nih.gov/geo/query/acc.cgi?acc=GSE125024.

## Supplementary Material

gkz584_Supplemental_File
